# The Half Yearly Abstract of Medical Sciences

**Published:** 1848-02

**Authors:** 


### PART III.—BIBLIOGRAPHICAL NOTICES.

### ARTICLE VIII.

*The Half Yearly Abstract of Medical Sciences.* Edited by W. H. Rankin, M. D. &c. &c., No. 6. July to January, 1838. (From the Publishers.)

The deservedly high reputation of this most excellent work is fully sustained in the number before us.

Its analytical digest of the contents of new works, and the reports which it gives on the progress of Medicine during the preceding six months, afford a greater amount of information, in a condensed form than can be obtained elsewhere for any thing like the trifling sum of 75 cents, the cost of a number.

No practitioner should neglect to supply himself with so valuable a source of information.

